# The Relationship of UGT2B15 Pharmacogenetics and Lorazepam for Anxiety

**DOI:** 10.7759/cureus.3133

**Published:** 2018-08-13

**Authors:** Nathan R Ching, Amna K Alzghari, Saeed K Alzghari

**Affiliations:** 1 Chemistry, Texas Christian University, Fort Worth, USA; 2 Pharmacy, CVS Health, Fort Worth, USA; 3 Pharmacotherapy, University of North Texas Health Sciences Center, Fort Worth, USA

**Keywords:** lorazepam, single nucleotide polymorphisms, anxiety, pharmacogenetics, ugt2b15

## Abstract

Anxiety affects over 260 million people worldwide. Benzodiazepines are a class of agents used in combination with other therapies for the management of anxiety. Lorazepam is a commonly prescribed benzodiazepine metabolized by uridine 5’-diphosphate-glucuronosyltransferases. Herein, we discuss recent findings regarding the pharmacogenetics of uridine 5’-diphosphate-glucuronosyltransferase 2B15 (UGT2B15), lorazepam, and its role in the treatment of anxiety.

## Editorial

Anxiety is a common mental disorder affecting 264 million people worldwide, which includes generalized anxiety disorder (GAD), phobias, post-traumatic stress disorder, panic disorder, obsessive-compulsive disorder, and social anxiety disorder [1]. In particular, the Diagnostic and Statistical Manual of Mental Disorders, 5th ed, characterizes the diagnostic criteria for GAD as follows: excessive anxiety and worry occurring more days than not for at least six months about a number of events or activities; the individual finds it difficult to control the worry; the anxiety is associated with three or more of the following symptoms that may include restlessness, being easily fatigued, difficulty concentrating, irritability, muscle tension, or sleep disturbance; the anxiety, worry, or physical symptoms cause clinically significant distress or impairment in social occupational, or other areas of functioning [2]. Lorazepam is a high potency benzodiazepine (BZD) used to augment first and second line therapies to relieve the acute symptoms of anxiety. Although BZDs are effective therapeutic agents due to their anxiolytic properties, drug interactions, adverse side effects, and risk of dependence prove to be a concern for clinicians when utilizing therapy [2-3]. BZDs target the same receptor as gamma-aminobutyric acid (GABA), a naturally occurring inhibitory neurotransmitter, acting on GABA-A receptors by inducing a conformational change in the receptor's chloride channel that hyperpolarizes the cell [3]. In turn, the inhibitory effect of GABA occurs throughout the central nervous system. Lorazepam’s metabolism is primarily by glucuronidation, bypassing phase I reactions by cytochrome P450.

Uridine 5’-diphosphate-glucuronosyltransferases are responsible for the glucuronidation of lorazepam. Of interest, a recent study showed that genetic polymorphisms in the uridine 5’-diphosphate-glucuronosyltransferase 2B15 (UGT2B15) enzyme might affect lorazepam’s metabolism. Chung et al. looked at the differences in the pharmacokinetics and pharmacodynamics of lorazepam in UGT2B15*1/*1 (wild type) and UGT2B15*2/*2 (homozygous variant) genotypes in human subjects [4]. Three metabolic states were tested: basal where metabolism was not inhibited or induced, inhibited metabolism by coadministration of valproate, and induced metabolism by rifampin pretreatment. The UGT2B15*2/*2 group showed 40% to 50% lower systemic clearance of lorazepam during basal state compared to the UGT2B15*1/*1 group. Additionally, in the induced and inhibited states, the UGT2B15*2/*2 group showed lower absolute values of clearance. In their analysis, the researchers concluded that the *UGT2B15* genotype accounted for 61% of the total variance of the systemic clearance of lorazepam, demonstrating a link between *UGT2B15* genetic polymorphism and the pharmacokinetic profile of lorazepam. However, evaluation of clinically relevant parameters including sedation and psychomotor performance showed no significant difference in the two groups when subjects received lorazepam in the basal state.

Although Chung et al. found a clinically insignificant relationship between *UGT2B15* polymorphisms and lorazepam pharmacokinetics [4], researchers took the opportunity to explore clinically relevant patient populations. Mijderwijk et al. investigated the relationship between lorazepam’s efficacy in lowering postoperative anxiety levels in surgery patients and *UGT2B15* genotype [5]. In this clinical trial, researchers evaluated anxiety levels before and after surgery in same-day surgery patients using the State part of the State-Trait Anxiety Inventory (STAI), analyzing the difference between the two measures as an indicator of anxiety reduction. Differences regarding post-operative anxiety scores varied between patient gender and initial premedication of lorazepam. In men with high preoperative anxiety scores, anxiety reduction in the UGT2B15*2/*2 group was much higher in the placebo group than the lorazepam group, whereas the lorazepam group showed greater anxiety reduction than the placebo group in UGT2B15*1/*1 and UGT2B15*1 /*2 (heterozygous variant) individuals. Interestingly enough, this relationship was the opposite in female patients, as females in the UGT2B15*2/*2 group showed greater anxiety reduction due to lorazepam premedication than placebo.

As shown in aforementioned studies, understanding the link between anxiety, lorazepam, and *UGT2B15 *genotype may help clinicians decide when to prescribe lorazepam in certain patient populations in the future. Testing for the *UGT2B15 *genotype could potentially be a part of the larger clinical picture focusing on patient-centered care pending further evidence and cost considerations. Future research should focus on other types of anxiety exploring the effect of the *UGT2B15* genotype on lorazepam.
